# Effect of age and isolated systolic or diastolic hypertension on target organ damage in non-dialysis patients with chronic kidney disease

**DOI:** 10.18632/aging.202609

**Published:** 2021-02-22

**Authors:** Yu Hao, Xue Li, Ye Zhu, Jianting Ke, Tanqi Lou, Man Li, Cheng Wang

**Affiliations:** 1 Division of Nephrology, Department of Medicine, The Fifth Affiliated Hospital Sun Yat-Sen University, Zhuhai 519000, Guangdong, China; 2 Guangdong Provincial Key Laboratory of Biomedical Imaging, The Fifth Affiliated Hospital Sun Yat-Sen University, Zhuhai 519000, Guangdong, China; 3 Division of Nephrology, Department of Medicine, Third Affiliated Hospital of Sun Yat-Sen University, Guangzhou 510630, Guangdong, China

**Keywords:** age, isolated hypertension, target organ damage, chronic kidney disease, ambulatory blood pressure monitoring

## Abstract

The aim of this study was to investigate associations between age-dependent variations in isolated systolic/diastolic hypertension (ISH/IDH) with target organ damage in chronic kidney disease (CKD). A cross-sectional study was conducted among 2,459 CKD patients with ambulatory blood pressure monitoring. Blood pressure was categorized into four groups: normotension, ISH, IDH, and systolic-diastolic hypertension. The outcome measurements were left ventricular mass index (LVMI), estimated glomerular filtration rate(eGFR), and urinary albumin creatinine ratio (ACR). Older patients (≥60-years-old) had a higher prevalence of ISH and a lower prevalence of IDH than younger patients (<60-years-old). In multivariate analysis, compared with the normotension group, younger patients with ISH were associated with higher LVMI (+14.4 g/m^2^), lower eGFR (−0.2 log units), and higher ACR (+0.5 log units); but younger patients with IDH were only associated with lower eGFR (−0.2 log units) and higher ACR (+0.4 log units). Among older patients, ISH was correlated with higher LVMI (+8.8 g/m^2^), lower eGFR (−0.2 log units), and higher ACR (+1.0 log units), whereas IDH was not associated with these renal/cardiovascular parameters. In conclusion, ISH was associated with a relatively high risk of target organ damage irrespective of age, whereas IDH was only correlated with renal injury in younger CKD patients.

## INTRODUCTION

Chronic kidney disease (CKD) is a major global public health problem. Even in the early stage, CKD patients have a higher risk of cardiovascular disease (CVD) [1, 2]. Although elevated blood pressure (BP) has long been established as an important risk factor for CVD in CKD patients [3], the risk conferred by hypertension subtypes including isolated systolic hypertension (ISH), isolated diastolic hypertension (IDH), and systolic-diastolic hypertension (SDH) [4, 5] has not been well examined. ISH is mostly related to increased arterial stiffness and IDH is associated with elevated vascular resistance in the arteriolar sector [6]. In the clinic, there has been a gradual shift from diastolic (D)BP to systolic (S)BP as the main predictor of cardiovascular risk with advanced age [7, 8]. Recent data from hypertensive patients and the general population have demonstrated age-related differences in the association of ISH and IDH with subclinical target organ damage. Their main findings were that ISH, rather than IDH, was related to cardiac damage regardless of age [9–11], and ISH conferred increased risk of diminished kidney function, which corresponded with increased serum creatinine among older participants [12]. In contrast, IDH was associated with a higher risk of renal damage such as albuminuria only in younger participants [4].

To the best of our knowledge, data on the correlation of ISH or IDH with subclinical cardiac damage in different age groups of CKD patients are scarce. Limited research [13] has been focused on the ability to predict increased risk of end-stage renal disease (ESRD) by ISH and IDH as determined by clinical BP measurements, but without age stratification. Additionally, ambulatory BP monitoring (ABPM) can substantially refine risk stratification [14], which can be applied to this research. It has been demonstrated that the presence of increased left ventricular mass index (LVMI) and albuminuria as well as reduced estimated glomerular filtration rate (eGFR) in patients with CKD is associated with worsening cardiovascular outcomes [3, 15]. Therefore, we enrolled 2459 CKD patients into this study with ABPM to explore associations between renal/cardiovascular parameters and ISH or IDH in different age groups.

## RESULTS

### BP types and baseline characteristics

A total of 2459 patients were included in this study. The mean age of participants was 46.8±14.9-years-old, and 1091 patients (44.4%) were women. The mean eGFR was 58.2 mL/min/1.73 m^2^. The prevalence of normotension (NM), ISH, IDH, and SDH among all patients were 38.3%, 7.6%, 15.7%, and 38.4%, respectively. Table 1 shows baseline characteristics of the patients divided into two age groups (<60- versus ≥60-years-old) and four BP types. There were 1,894 patients in the group of younger individuals (<60-years-old) and 565 patients in the group of older participants. ISH, IDH and SDH were found in 4.0%, 17.8%, and 37.2% of younger patients and in 19.8%, 8.7%, and 42.3% of older patients, respectively. The proportion of patients in each BP group was significantly different between the two age groups (all P<0.05). In pairwise comparisons, eGFR and ACR among the ISH and SDH patients showed no significant difference, but were higher than in the IDH and NM patients among both the younger and older cohorts. We also observed a similar trend for LVMI among the elderly patients when divided in to the BP groups; however, for the younger cohort, LVMI was highest in the SDH group followed by the ISH, IDH, and NM groups (all P<0.05). In the comparison between the two age groups, among those in the normotension group, elderly patients had higher LVMI levels than younger patients (*P*<0.01). The eGFR levels were significantly lower in elderly patients compared with younger patients, except in the ISH and SDH groups.

**Table 1:** Baseline characteristics according to age and blood pressure groups.

	**Age < 60 years**				**Age ≥ 60 years**			
**Parameter**	**NM** **n=777 (41.0%)**	**ISH** **n=76 (4.0%)**	**IDH** **n=336 (17.8%)**	**SDH** **n=705 (37.2%)**	**NM** **n=165 (29.2%)**	**ISH** **n=112 (19.8%)**	**IDH** **n=49 (8.7%)**	**SDH** **n=239 (42.3%)**
**Demographics and Past Medical History**								
Age, years	37.4±11.6	43.1±12.8^*^	43.4±10.5^*^	43.4±10.7^*^	66.3±4.4	68.3±4.9^*^	64.6±3.6^†^	66.0±4.7^†^
Female, No. (%)	428(55.1)	21(27.6)^*^	128(38.1)^*^	274(38.9)^*^	83(50.3)	48(42.9)	20(40.8)	89(37.2)
Current smoker, No. (%)	127(16.3)	25(32.9)^*^	88(26.2)^*^	178(25.2)^*^	34(20.6)	28(25.0)	14(28.6)	62(25.9)
Alcohol intake, No. (%)	106(13.7)	13(17.1)	101(30.1)^*^	120(17.0)^‡^	25(15.2)	13(11.6)	9(18.4)	40(16.7)
Diabetes mellitus, No. (%)	53(6.8)	27(35.5)^*^	47(14.0)^*†^	154(21.8)^*†‡^	60(36.4)	59(52.7)^*^	20(40.8)	104(43.5)
Hyperlipidemia, No. (%)	118(15.2)	12(15.8)	81(24.1)^*^	159(22.6)^*^	38(23.0)	30(26.8)	11(22.4)	60(25.1)
CVD history, No. (%)	29(3.7)	4(5.3)	31(9.2)^*^	93(13.2)^*^	33(20.0)	18(16.1)	13(26.5)	61(25.5)
Hypertension, No. (%)	135(17.4)	38(50.0)^*^	179(53.3)^*^	503(71.3)^*†‡^	89(53.9)	86(76.8)^*^	33(67.3)	207(86.6)^*‡^
Antihypertension drugs, No. (%)	420(54.1)	59(77.6)^*^	245(72.9)^*^	620(87.9)^*‡^	109(66.1)	98(87.5)^*^	35(71.4)	210(87.9)^*‡^
RAS blockade, No. (%)	345(44.4)	36(47.4)	144(42.9)	334(47.4)	57(34.5)	54(48.2)	16(32.7)	90(37.7)
**etiology of CKD**								
Primary glomerulonephritis, No. (%)	552(71.0)	38(50.0)^*^	215(64.0)	445(63.1)^*^	74(44.8)	57(50.9)	22(44.9)	106(44.3)
Diabetic nephropathy, No. (%)	17(2.2)	20(26.3)^*^	12(3.6)^†^	95((13.5)^*†‡^	22(13.4)	28(25.0)	6(12.2)	52(21.8)
Hypertensive nephropathy, No. (%)	10(1.3)	0(0.0)	21(6.2)^*^	36(5.1)^*^	17(10.3)	6(5.3)	6(12.2)	28(11.7)
Other causes, No. (%)	198(25.5)	18(23.7)	88(26.2)	129(18.3)^*‡^	52(31.5)	21(18.8)	15(30.6)	53(22.2)
**Physical Examination**								
Body mass index, kg/m^2^	22.7±3.7	23.5±3.6	24.2±3.6^*^	24.0±3.7^*^	23.2±3.6	24.2±2.8	24.6±2.9	24.4±3.9^*^
24 h-SBP, mm Hg	112.1±8.4	137.7±8.9^*^	122.2±4.7^*†^	146.2±12.4^*†‡^	117.6±8.4	141.1±8.9^*^	124.1±4.3^*†^	149.0±12.7^*†‡^
24 h-DBP, mm Hg	70.6±5.6	75.7±3.3^*^	84.2±3.8^*†^	92.5±8.3^*†‡^	71.5±5.8	74.5±3.8^*^	83.9±3.8^*†^	87.7±6.1^*†‡^
**Laboratory Values**								
Serum creatinine, umol/L	76.0(60.0-107.5)	190.9(87.5-596.5)^*^	109.5(75.0-160.0)^*†^	209.0(99.0-576.0)^*‡^	122.0(80.5-223.0)	189.0(106.6-500.0)^*^	119.0(87.0-204.0)	229.0(109.0-58.9)^*‡^
Serum uric acid, mmol/L	403.1±127.2	503.7±169.0^*^	443.4±140.5^*†^	491.0±139.7^*‡^	441.1±136.5	477.9±135.3	422.9±118.0	468.8±123.4
Serum fasting glucose, mmol/L	5.0±1.9	5.6±2.0	5.2±1.3	5.5±2.2^*^	6.1±2.3	6.1±2.3	5.9±2.1	5.7±1.8
Triglyceride, mmol/L	1.3(0.9-1.9)	1.4(0.9-2.1)	1.7(1.2-2.3)^*^	1.7(1.2-2.4)^*^	1.6(1.0-2.2)	1.3(1.0-1.9)	1.6(1.0-2.0)	1.5(1.1-2.3)
Total cholesterol, mmol/L	5.0(4.0-6.0)	4.9(3.4-6.2)	5.1(4.2-6.0)	4.9(4.1-6.2)	4.7(3.7-6.0)	4.8(3.9-5.9)	4.7(3.8-6.1)	4.9(3.9-6.0)
HDL-C, mmol/L	1.3±0.5	1.2±0.5	1.1±0.4^*^	1.1±0.4^*^	1.2±0.4	1.1±0.3	1.1±0.3	1.1±0.4
LDL-C, mmol/L	3.4±1.9	3.1±1.8	3.1±1.1	3.3±1.7	2.9±1.3	3.0±1.2	2.8±1.2	3.0±1.3
Serum albumin, g/L	37.1±8.2	34.5±7.7	38.7±6.5^*†^	35.6±7.4^*‡^	37.2±6.4	36.1±5.9	37.6±7.0	36.3±6.6
Hemoglobin, g/L	128.6±23.3	105.6±30.6^*^	132.4±24.8^†^	112.3±30.2^*‡^	115.2±23.9	103.5±26.2^*^	122.7±22.5^†^	109.3±24.7^‡^
Serum calcium, mmol/L	2.1(2.0-2.3)	2.1(2.0-2.2)	2.2(2.1-2.3)	2.1(2.0-2.3)	2.2(2.1-2.2)	2.2(2.1-2.3)	2.1(2.0-2.2)	2.1(2.0-2.2)
Serum phosphate, mmol/L	1.1(1.0-1.3)	1.3(1.1-1.6)^*^	1.1(0.9-1.2)^†^	1.3(1.1-1.6)^*‡^	1.2(1.0-1.3)	1.2(1.1-1.4)	1.0(0.9-1.2)^†^	1.2(1.0-1.5)^*‡^
iPTH, pmol/L	5.5(3.5-9.4)	8.2(3.5-21.9)^*^	6.1(4.0-9.4)	10.2(5.4-21.4)^*‡^	7.2(4.4-11.9)	11.6(5.9-20.8)^*^	6.5(4.1-10.4)^†^	10.8(5.7-22.6)^*‡^
eGFR, mL/min/1.73 m^2^	99.0(67.4-116.1)	30.8(8.0-93.2)^*^	68.7(40.8-100.7)^*†^	29.8(7.9-74.4)^*‡^	51.0(21.5-81.7)^#^	24.7(9.1-58.4)^*^	53.6(26.4-73.1)^†#^	21.4(8.4-51.3)^*‡^
Albumin creatinine ratio, mg/g	300.4 (40.4-986.3)	1253.4 (276.1-2436.0)^*^	449.2 (73.6-1210.7)^†^	1131.1 (379.5-2319.7)^*‡^	222.1 (22.5-977.0)	1114.4 (410.1-2009.9)^*^	277.7 (22.1-1202.7)^†^	977.0 (205.5-1891.7)^*‡^
Left ventricular mass index, g/m2	82.5±19.5	115.6±29.3^*^	88.7±21.5^*†^	115.0±36.6^*‡^	99.8±27.5^#^	117.0±29.2^*^	94.7±23.0^†^	113.8±31.3^*‡^

Data are presented as numbers and percentages, means and standard deviations, or medians and quartile ranges. NM, normotension; ISH, isolated systolic hypertension; IDH, isolated diastolic hypertension; SDH, systolic and diastolic hypertension; CVD, cardiovascular disease; RAS blockade, renin-angiotensin system blockade; SBP, systolic blood pressure; DBP, diastolic blood pressure; HDL-C, high-density lipoprotein cholesterol; LDL-C, low-density lipoprotein cholesterol; iPTH, intact parathyroid hormone; eGFR, estimated glomerular filtration rate; CKD, chronic kidney disease. * compared with normotension, *P* <0.01 for continuous variables, *P* <0.008 for categorical variables; † compared with isolated systolic hypertension, *P* <0.01 for continuous variables, *P* <0.008 for categorical variables; ‡ compared with isolated diastolic hypertension, *P* <0.01 for continuous variables, *P* <0.008 for categorical variables; # represents a comparison with patients under 60 years old with the same blood pressure type, *P* <0.01.

### BP types in different CKD stages

The prevalence of hypertensive subtypes in different stages of CKD is shown in Table 2. The overall rate of NM was 38.3%, which decreased with advancement of CKD (from 63.7% in stage 1 to 15.7% in stage 5). There was a stepwise increase in the prevalence of ISH and SDH by CKD stage (ISH: 3.7% and 13.4% in stages 1 and 5, respectively; SDH: 17.4% and 63.6% in stages 1 and 5, respectively). In contrast, the prevalence of IDH increased from 15.2% to 22.4% from stages 1 to 3, respectively, and then decreased to 12.8% and 7.3% in stages 4 and 5, respectively. We found a linear trend in the proportion of BP types across the CKD stages in all four BP types (P<0.05). These trends were consistent between younger and older patients except for the proportion of IDH in older patients, which began to decline at stage 3.

**Table 2:** Blood pressure types in different CKD stages.

**N (%)**	**CKD 1**	**CKD 2**	**CKD 3**	**CKD 4**	**CKD 5**	***P-trend* value**
Total	771	437	450	235	566	
NM	491(63.7)	185(42.3)	123(27.3)	54(23.0)	89(15.7)	<0.01
ISH	29(3.7)	25(5.7)	35(7.8)	23(9.8)	76(13.4)	<0.01
IDH	117(15.2)	96(22.0)	101(22.4)	30(12.8)	41(7.3)	<0.01
SDH	134(17.4)	131(30.0)	191(42.5)	128(54.4)	360(63.6)	<0.01
Age < 60 years	714	341	302	141	396	
NM	462(64.7)	150(44.0)	77(25.5)	29(20.6)	59(14.9)	<0.01
ISH	20(2.8)	9(2.6)	9(3.0)	6(4.2)	32(8.1)	<0.01
IDH	112(15.7)	83(24.4)	83(27.5)	22(15.6)	36(9.1)	0.01
SDH	120(16.8)	99(29.0)	133(44.0)	84(59.6)	269(67.9)	<0.01
Age ≥ 60 years	57	96	148	94	170	
NM	29(50.9)	35(36.5)	46(31.1)	25(26.6)	30(17.7)	<0.01
ISH	9(15.8)	16(16.7)	26(17.5)	17(18.1)	44(25.9)	0.04
IDH	5(8.8)	13(13.5)	18(12.2)	8(8.5)	5(2.9)	<0.01
SDH	14(24.5)	32(33.3)	58(39.2)	44(46.8)	91(53.5)	<0.01

NM, normotension; ISH, isolated systolic hypertension; IDH, isolated diastolic hypertension; SDH, systolic and diastolic hypertension; CKD, chronic kidney disease.

### Associations between age, BP types, and sub-clinical target organ damage in CKD patients

In univariate analyses, ISH, IDH, and SDH were correlated with higher LVMI and ACR, and lower eGFR (Model 1 in Table 3) when each was compared with NM. After adjusting for older age (≥60 years), these associations remained statistically significant (Model 2 in Table 3). Older patients had higher LVMI (+6.1 g/m^2^) and lower eGFR (−0.3 log units) and lower ACR (−0.3 log units) than younger patients. To further adjust for other confounding risk factors (Model 3 in Table 3), we compared NM with both ISH and SDH and found that the latter two were associated with higher LVMI without age modification. All of the hypertension subtypes including ISH, IDH, and SDH were associated with lower eGFR and higher ACR (*P*<0.05).

**Table 3:** Multivariable linear regression analysis for blood pressure types, age and left ventricular mass index (LVMI), the Log (estimated glomerular filtration rate) (eGFR), and the Log (albumin creatinine ratio) (ACR).

	**LVMI**			**Log (eGFR)**			**Log (ACR)**		
	**Model 1**	**Model 2**	**Model 3**	**Model 1**	**Model 2**	**Model 3**	**Model 1**	**Model 2**	**Model 3**
NM	reference	reference	reference	reference	reference	reference	reference	reference	reference
ISH	30.9(26.5~35.4)*	28.4(23.8~32.9)*	13.2(9.0~17.4)*	-1.0(-1.1~-0.8)*	-0.9(-1.0~-0.7)*	-0.2(-0.3~-0.1)*	1.3(1.0~1.7)*	1.5(1.2~1.8)*	0.7(0.5~1.0)*
IDH	3.9(0.6~7.3)*	4.2(0.9~7.6)*	-0.7(-3.7~2.3)	-0.2(-0.3~-0.1)*	-0.2(-0.4~-0.1)*	-0.2(-0.3~-0.2)*	0.3(0.1~0.5)*	0.3(0.0~0.5)*	0.3(0.1~0.5)*
SDH	29.2(26.6~31.8)*	28.7(26.2~31.3)*	11.9(9.3~14.5)*	-1.0(-1.1~-1.0)*	-1.0(-1.1~-0.9)*	-0.4(-0.5~-0.4)*	1.3(1.1~1.4)*	1.3(1.1~1.5)*	0.5(0.3~0.7)*
Age	-	6.1(3.4~8.9)*	-0.2(-2.8~2.4)	-	-0.3(-0.4~-0.2)*	-0.2(-0.3~-0.2)*	-	-0.3(-0.5~-0.1)*	-0.5(-0.6~-0.3)*

Data are presented as unstandardized coefficients beta (95% CIs). Model 1: only contains ISH, IDH, SDH; model 2: contains ISH, IDH, SDH and age (0= age <60 years, 1= age ≥ 60 years); model 3: contains variables of model 2 and additional adjustment variables of LVMI include gender, BMI, current smoker, diabetes mellitus, CVD history, antihypertensive drugs, hemoglobin, uric acid, serum fasting glucose, HDL-C, LDL-C, serum albumin, serum phosphate, iPTH, eGFR; additional adjustment variables of Log (eGFR) include gender, BMI, alcohol intake, diabetes mellitus, hyperlipidemia, CVD history, antihypertensive drugs, hemoglobin, uric acid, HDL-C, LDL-C, serum phosphate, iPTH; additional adjustment variables of Log (ACR) include gender, alcohol intake, diabetes mellitus, CVD history, antihypertensive drugs, hemoglobin, uric acid, triglyceride, total cholesterol, HDL-C, LDL-C, serum albumin, serum phosphate, iPTH, eGFR.

BMI, body mass index; CVD, cardiovascular disease; HDL-C, high-density lipoprotein cholesterol; LDL-C, low-density lipoprotein cholesterol; iPTH, intact parathyroid hormone; NM, normotension; ISH, isolated systolic hypertension; IDH, isolated diastolic hypertension; SDH, systolic and diastolic hypertension; LVMI, left ventricular mass index; eGFR, estimated glomerular filtration rate; ACR, albumin creatinine ratio. * *P* <0.05.

### Association of BP types and sub-clinical target organ damage in different age groups

**Figure 1:**
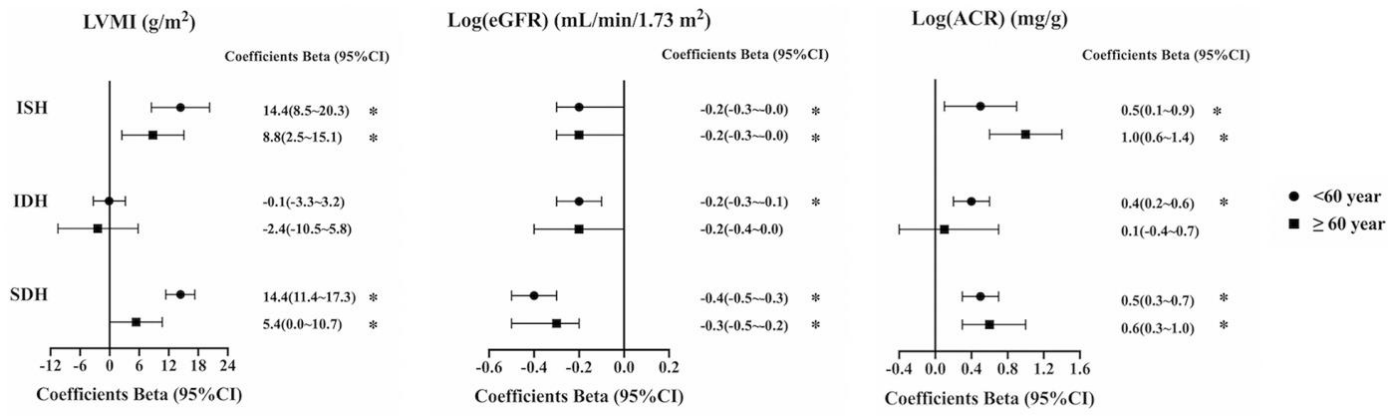
**Relationship between left ventricular mass index (LVMI), the Log (estimated glomerular filtration rate) (eGFR), and the Log (albumin creatinine ratio) (ACR) and blood pressure types in different age groups.** In patients under 60 years old, adjusted variables for LVMI include gender, age, BMI, current smoker, diabetes mellitus, CVD history, antihypertensive drugs, hemoglobin, uric acid, HDL-C, LDL-C, serum albumin, serum phosphate, iPTH, eGFR, ISH, IDH, SDH; adjusted variables for Log (eGFR) include gender, age, BMI, current smoker, alcohol intake, diabetes mellitus, hyperlipidemia, CVD history, antihypertensive drugs, hemoglobin, uric acid, total cholesterol, HDL-C, LDL-C, serum phosphate, iPTH, ISH, IDH, SDH; adjusted variables for Log (ACR) include gender, age, alcohol intake, diabetes mellitus, antihypertensive drugs, hemoglobin, uric acid, triglyceride, total cholesterol, HDL-C, LDL-C, serum albumin, serum phosphate, iPTH, eGFR, ISH, IDH, SDH. In patients at and above 60 years old, adjusted variables for LVMI include gender, current smoker, hyperlipidemia, CVD history, antihypertensive drugs, hemoglobin, uric acid, HDL-C, serum phosphate, iPTH, eGFR, ISH, IDH, SDH; adjusted variables for Log (eGFR) include gender, age, BMI, hyperlipidemia, antihypertensive drugs, hemoglobin, uric acid, triglyceride, HDL-C, LDL-C, serum phosphate, iPTH, ISH, IDH, SDH; adjusted variables for Log (ACR) include gender, diabetes mellitus, antihypertensive drugs, hemoglobin, HDL-C, LDL-C, serum albumin, serum calcium, serum phosphate, iPTH, eGFR, ISH, IDH, SDH. BMI, body mass index; CVD, cardiovascular disease; HDL-C, high-density lipoprotein cholesterol; LDL-C, low-density lipoprotein cholesterol; iPTH, intact parathyroid hormone; NM, normotension; ISH, isolated systolic hypertension; IDH, isolated diastolic hypertension; SDH, systolic and diastolic hypertension; LVMI, left ventricular mass index; eGFR, estimated glomerular filtration rate; ACR, albumin creatinine ratio. * *P* <0.05.

After stratifying the two age groups, we found that ISH was significantly associated with higher LVMI compared with the NM group in both younger and older patients (+14.4 g/m^2^ and +8.8 g/m^2^, respectively, both *P*<0.05). SDH was also significantly associated with higher LVMI in both younger and older patients (+14.4 g/m^2^ and +5.4 g/m^2^, respectively, both *P*<0.05). There was no relationship between IDH and LVMI in either age group (Figure 1).

Compared with NM, ISH was significantly associated with lower eGFR in both younger and older patients (−0.2 log units and −0.2 log units, respectively, both *P*<0.05). SDH was also significantly associated with lower eGFR in both younger and older patients (−0.4 log units and −0.3 log units, respectively, both *P*<0.05). IDH was only negatively associated with eGFR among younger patients (−0.2 log units, *P*<0.05) (Figure 1).

Compared with NM, ISH was significantly associated with higher ACR in both younger and older patients (+0.5 log units and +1.0 log units, respectively, both *P*<0.05). SDH was also significantly associated with higher ACR in both younger and older patients (+0.5 log units and +0.6 log units, respectively, both *P*<0.05). IDH was only positively correlated with ACR among young patients (+0.4 log units, *P*<0.05) (Figure 1).

## DISCUSSION

In this cross-sectional study, we studied associations between distinct types of ambulatory hypertension and several markers of subclinical target organ damage in CKD patients of different ages. The two most clinically important findings of our study were as follows: (1) in younger patients, using NM as a reference, ISH and SDH had higher LVMI and ACR, and lower eGFR, while only IDH correlated with renal damage; (2) in older patients, only ISH and SDH were correlated with these renal/cardiovascular parameters. These data suggest that age plays a role in the target organ damage from ISH and IDH in CKD patients, and therefore special but different attention should be paid to CKD patients with isolated hypertension in different ages.

Based on the current pathophysiological understanding, the hypertension subtypes are manifested by singular elevations in SBP or DBP, which may reflect unique biological processes that are closely related to age, because aging causes structural changes to blood vessels [16]. The mechanisms of ISH and IDH among young adults may differ. Increased aortic stiffness, exaggerated pulse pressure amplification from central to peripheral arteries, and stroke volume seem to contribute more to ISH individuals [17], whereas higher systemic vascular resistance is the major contributor to high DBP [18]. With increasing age and progressive aortic stiffness, ISH becomes the most common form of hypertension in the elderly, and due to the loss of Windkessel function and increased diastolic runoff, there is reduced prevalence of IDH [19–21]. Therefore, we investigated the prevalence of ISH and IDH in CKD patients of different ages for the first time. Among all CKD patients in this study, ISH accounted for 7.6% and IDH accounted for 15.7%. Meanwhile, compared with the younger patients, older patients had a higher prevalence of ISH and a lower prevalence of IDH, which was consistent with a previous study [22]. These data show that age is an important factor that affects the type of hypertension. Previous studies have confirmed the singular effects of ISH and IDH on the increased risk of target organ damage in different aged hypertensive adults, with a growing awareness that ISH and IDH may have distinct age-related clinical implications. However, no study had clarified these associations in CKD patients, which is important, as these patients have a high cardiovascular risk.

Here, we enrolled 2459 CKD patients with ABPM and first explored associations between target organ damage and isolated systolic or diastolic hypertension in different age groups. We found that ISH and SDH had higher risks of target organ damage irrespective of age or target organ, whereas IDH was only associated with renal damage in younger patients. These results suggest that age should be taken into account when assessing the cardiovascular risk of isolated hypertension in non-dialysis CKD patients.

Among the markers of cardiac damage, LVMI is a powerful and well-established predictor of CVD in CKD patients [15] that has a pathophysiological relationship with ISH. Most ISH cases are caused by the reduced elasticity and compliance of large arteries that results from age. Elevated BP itself can promote further arterial stiffening and impair endothelium-dependent vasodilatation [23]. To maintain appropriate cardiac output against increased afterload and decreased compliance from a stiffened arterial tree, the left ventricle stiffens and hypertrophies [22]. Extensive cross-sectional studies using echocardiography have found that older ISH patients have abnormal left ventricular mass and left ventricular geometry [24, 25]. Thus, it was not a surprise to find that ISH was accompanied by a high risk of cardiac damage in CKD patients, which manifested as a relatively high level of LVMI and was consistent with previous studies [26–28]. We also found that such a correlation existed in all age groups of CKD patients. We speculate that early identification and proper management of ISH might help to reduce the cardiac injury in CKD patients irrespective of age.

In addition to heart damage, both aging and hypertension are associated with decreased renal function [29, 30]. Our study found that the effect of ISH and IDH on renal damage was age-related. In younger patients, both ISH and IDH were associated with renal parameters, while ISH, rather than IDH was correlated with kidney damage in older patients. These results were consistent with previous studies in hypertension patients. As a previous study showed, younger patients (35–57-years-old) with systolic hypertension had a higher prevalence of ESRD, regardless of DBP levels, while diastolic hypertension was associated with an increased risk of ESRD in patients with normal SBP [31]. Another study on the hypertension population suggested that 24 h DBP and IDH only relate to the urinary albumin creatinine ratio in patients below 55-years-old [4]. ISH gradually became the dominant risk factor for renal damage, which may be attributed to stiffening of the arterial walls with aging. Several lines of evidence have suggested that increased aortic stiffness promotes deterioration of renal function with albuminuria and decreased eGFR [32–34]. In our study, the increased prevalence of ISH and SDH in advanced CKD stages dramatically contributed to the gradual decrease in BP control rate, especially in older patients. Just as a high BP accelerates the age-associated decline in eGFR [34], this may also support the positive correlation between ISH and renal damage in older patients. Further and extensive studies are needed to explain the above-mentioned phenomenon. However, proper management of ISH and IDH in younger CKD patients might help to improve albuminuria and the reduced eGFR, while more attention should be paid to ISH in older CKD patients.

This study has several strengths. First, to the best of our knowledge, this is the first study to investigate the impact of age on correlations between isolated hypertension and target organ damage in non-dialysis CKD patients in China. Identifying non-benign BP types and further optimizing the cardiovascular risk assessment for CKD patients are particularly important. Second, all included patients had comprehensive assessments, and the cohort size was large. Third, we measured BP by 24-h ambulatory monitoring, given that ABPM is now considered a keystone in hypertension management [35]. ISH and IDH have been associated with a high prevalence of “white coat hypertension,” even higher than SDH in all age groups [36], which can be avoided by ABPM. Our study also needs to be interpreted with the recognition of its shortcomings. Since this is an observational study, only associations, but no cause-and-effect relationships can be established from it. Moreover, extrapolations of the results to other racial groups should be made with care due to the fact that all of the participants were Chinese. In addition, due to limited sample size, this study did not explore relationships between BP types and target organ injury in different causes of CKD. A larger sample cohort in a multicenter prospective study is needed in the future. Specifically, some participants in our study were on one or more antihypertensive medications, mostly RAS blockers, which could potentially impact our observations. Finally, we found a negative association between age and ACR, which is contrary to the Framingham et al. study in the general population [37]. The potential reasons are as follows: (1) our study focused on Chinese CKD patients, which is different from the Framingham study; (2) the negative correlation between age and ACR may be confounded by the causes of CKD (Supplementary Table 1), which may need more investigation in the future.

In conclusion, our findings highlight the age-specific effects of different isolated hypertension types on cardiac and renal damage in CKD patients and have potential clinical implications. ISH was generally related to cardiac and renal damage without age modifications, while IDH was only harmful to renal function, and then mainly in younger patients. Considering that ISH and IDH, as intermediate phenotypes with unpredictable development, have a high probability of transitioning to SDH [17, 38], the long-term injury of ISH and IDH may be tremendous. Therefore, timely attention and management may bring clinical benefits. Future clinical research is needed to examine whether prompt aggressive therapy for isolated hypertension before the onset of target organ damage can reduce cardiovascular risk.

## MATERIALS AND METHODS

### Study population

The study protocol was approved by the ethics committee of our hospitals, and was approved by the Institutional Review Board. All patients gave their written informed consent to the use of data for scientific purposes.

A total of 2850 CKD inpatients aged 18 to 75 years and completed ABPM for this cross-sectional study. We excluded 391 participants according to the following criteria: dialysis or transplant, changes in the estimated glomerular filtration rate (eGFR) >30% in the previous 3 months; pregnancy; atrial fibrillation; inadequate ABPM readings; night work or shift-work employment; inability to communicate and comply with all of the study requirements. Finally, a total of 2459 CKD patients were included into the current analysis. In terms of causes of renal disease, 1509 patients had primary glomerulonephritis; 252 cases had diabetic nephropathy; 124 subjects had hypertensive nephropathy; and 574 patients had other causes of renal disease.

### Blood pressure measurement

Ambulatory blood pressure monitoring was performed with the automated measurements programmed at 15-minute intervals during the daytime and 30-minute intervals at night as previously [39, 40]. The valid measurements had to fulfill prespecified quality criteria, including the successful recording of a minimum 20 valid daytime and at least 7 valid nighttime measurements, and at least 70% of the expected 24-hour readings [35, 41]. Day and night periods were defined according to sleeping and waking times reported by the patient.

Using mean 24-hour ambulatory thresholds [35], regardless of antihypertensive drugs, normotension (NM) was defined as SBP <130 mmHg and DBP <80 mmHg; ISH was defined as SBP ≥130 mmHg and DBP <80 mmHg; IDH was defined as SBP <130 mmHg and DBP ≥80 mmHg; And SDH was defined as SBP ≥130 mmHg and DBP ≥80 mmHg.

### Cardiac assessment

Echocardiography was performed by two trained cardiologists, according to the recommendations of the American Society of Echocardiography and the European Association of Cardiovascular Imaging [42]. Details of left ventricular mass (LVM) measurement and calculation have been previously reported [39], LVMI was the LVM standardized by body surface area [43].

### Renal assessment

An isotope dilution mass spectrometry–traceable methodology was utilized to determine serum creatinine, and eGFR was estimated using the CKD-EPI (CKD–Epidemiology Collaboration) formula. According to Kidney Disease Improving Global Outcomes (KDIGO) [3], based on eGFR levels, CKD patients were divided into five stages (1, 2, 3, 4, 5).

As recommended in KDIGO guidelines, we preferred urine albumin creatinine ratio (ACR) as the measure of albuminuria. A first morning urine sample was collected on the day of ambulatory blood pressure measurement, and the concentration of urinary albumin and creatinine were measured by immunoturbidimetry in the central laboratory.

### Other measurements

Patient data including sociodemographic and clinical characteristics, medical history, and current therapy were obtained from interviews and physical examinations at the initial study visit and from clinical records. Body mass index was weight in kilograms divided by the height in meters squared. The definitions of Diabetes mellitus, Hyperlipidemia, CVD history have been previously reported [39]. In addition, A fasting blood sample was collected to measure hemoglobin, albumin, calcium, phosphorus, intact parathyroid hormone, serum fasting glucose, cholesterol, triglycerides, high-density lipoprotein cholesterol, low-density lipoprotein-cholesterol, homocysteine, uric acid, Scr, blood urea nitrogen, which were measured using a 7180 Biochemistry Auto-analyzer (Hitachi, Tokyo, Japan) in the central laboratory.

### Statistical analysis

Data were tested for normal distribution using the Kolmogorov–Smirnov test. Descriptive statistics are presented as the mean ± standard deviation (SD) for normally distributed variables and as the median (interquartile range) for non-normally distributed variables. Frequency and percentage were used for categorical variables. Comparisons among the BP groups were performed using ANOVA or nonparametric tests for continuous variables and the χ2 test for categorical variables. The Bonferroni method was used for post hoc pairwise comparisons. A multiple linear regression model was used to analyze the cross-sectional association of age and BP types with parameters of subclinical target organ damage (LVMI, Log eGFR, Log ACR) before and after adjusting for other significant variables from the univariate linear regression analyses. Associations between age and the BP types were analyzed in the multivariate adjusted model. After stratification for age (<60- versus ≥60-years-old), multiple linear regression models were employed to study correlations between BP types and subclinical target organ damage parameters in the different age groups. In the graphs, the non-standardized coefficient beta-values (±95% confidence intervals [CIs]) are given, which correspond to the quantitative difference between each BP group and NM as the reference group. *P* values <0.05 were considered statistically significant. All statistical analyses were performed using SPSS version 25 (IBM Corp., Armonk, NY, USA) and R Version 3.6.0. Graphs were generated with GraphPad Prism 8 (GraphPad Software, Inc., San Diego, CA, USA).

## Supplementary Material

Supplementary Table 1

## References

[1] Matsushita K, van der Velde M, Astor BC, Woodward M, Levey AS, de Jong PE, Coresh J, Gansevoort RT, and Chronic Kidney Disease Prognosis Consortium. Association of estimated glomerular filtration rate and albuminuria with all-cause and cardiovascular mortality in general population cohorts: a collaborative meta-analysis. Lancet. 2010; 375:2073–81. 10.1016/S0140-6736(10)60674-5 20483451 PMC3993088

[2] Gansevoort RT, Correa-Rotter R, Hemmelgarn BR, Jafar TH, Heerspink HJ, Mann JF, Matsushita K, Wen CP. Chronic kidney disease and cardiovascular risk: epidemiology, mechanisms, and prevention. Lancet. 2013; 382:339–52. 10.1016/S0140-6736(13)60595-4 23727170

[3] Inker LA, Astor BC, Fox CH, Isakova T, Lash JP, Peralta CA, Kurella Tamura M, Feldman HI. KDOQI US commentary on the 2012 KDIGO clinical practice guideline for the evaluation and management of CKD. Am J Kidney Dis. 2014; 63:713–35. 10.1053/j.ajkd.2014.01.416 24647050

[4] Wei FF, Li Y, Zhang L, Xu TY, Ding FH, Staessen JA, Wang JG. Association of target organ damage with 24-hour systolic and diastolic blood pressure levels and hypertension subtypes in untreated Chinese. Hypertension. 2014; 63:222–28. 10.1161/HYPERTENSIONAHA.113.01940 24246384

[5] McEvoy JW, Daya N, Rahman F, Hoogeveen RC, Blumenthal RS, Shah AM, Ballantyne CM, Coresh J, Selvin E. Association of isolated diastolic hypertension as defined by the 2017 ACC/AHA blood pressure guideline with incident cardiovascular outcomes. JAMA. 2020; 323:329–38. 10.1001/jama.2019.21402 31990314 PMC6990938

[6] Asgari S, Khalili D, Mehrabi Y, Kazempour-Ardebili S, Azizi F, Hadaegh F. Incidence and risk factors of isolated systolic and diastolic hypertension: a 10 year follow-up of the tehran lipids and glucose study. Blood Press. 2016; 25:177–83. 10.3109/08037051.2015.1116221 26643588

[7] Kannel WB, Gordon T, Schwartz MJ. Systolic versus diastolic blood pressure and risk of coronary heart disease. The Framingham study. Am J Cardiol. 1971; 27:335–46. 10.1016/0002-9149(71)90428-0 5572576

[8] Franklin SS, Larson MG, Khan SA, Wong ND, Leip EP, Kannel WB, Levy D. Does the relation of blood pressure to coronary heart disease risk change with aging? The Framingham Heart Study. Circulation. 2001; 103:1245–49. 10.1161/01.cir.103.9.1245 11238268

[9] McEvoy JW, Chen Y, Nambi V, Ballantyne CM, Sharrett AR, Appel LJ, Post WS, Blumenthal RS, Matsushita K, Selvin E. High-sensitivity cardiac troponin T and risk of hypertension. Circulation. 2015; 132:825–33. 10.1161/CIRCULATIONAHA.114.014364 26152706 PMC4558242

[10] Manios E, Michas F, Stamatelopoulos K, Koroboki E, Stellos K, Tsouma I, Vemmos K, Zakopoulos N. Association of isolated systolic, isolated diastolic, and systolic-diastolic masked hypertension with carotid artery intima-media thickness. J Clin Hypertens (Greenwich). 2015; 17:22–26. 10.1111/jch.12430 25329435 PMC8031861

[11] McEvoy JW, Chen Y, Rawlings A, Hoogeveen RC, Ballantyne CM, Blumenthal RS, Coresh J, Selvin E. Diastolic blood pressure, subclinical myocardial damage, and cardiac events: implications for blood pressure control. J Am Coll Cardiol. 2016; 68:1713–22. 10.1016/j.jacc.2016.07.754 27590090 PMC5089057

[12] Young JH, Klag MJ, Muntner P, Whyte JL, Pahor M, Coresh J. Blood pressure and decline in kidney function: findings from the Systolic Hypertension in the Elderly Program (SHEP). J Am Soc Nephrol. 2002; 13:2776–82. 10.1097/01.asn.0000031805.09178.37 12397049

[13] Peralta CA, Norris KC, Li S, Chang TI, Tamura MK, Jolly SE, Bakris G, McCullough PA, Shlipak M, and KEEP Investigators. Blood pressure components and end-stage renal disease in persons with chronic kidney disease: the kidney early evaluation program (KEEP). Arch Intern Med. 2012; 172:41–47. 10.1001/archinternmed.2011.619 22232147 PMC3417125

[14] Kelly TN, Gu D, Chen J, Huang JF, Chen JC, Duan X, Wu X, Yau CL, Whelton PK, He J. Hypertension subtype and risk of cardiovascular disease in Chinese adults. Circulation. 2008; 118:1558–66. 10.1161/CIRCULATIONAHA.107.723593 18809800 PMC2735390

[15] Eckardt KU, Scherhag A, Macdougall IC, Tsakiris D, Clyne N, Locatelli F, Zaug MF, Burger HU, Drueke TB. Left ventricular geometry predicts cardiovascular outcomes associated with anemia correction in CKD. J Am Soc Nephrol. 2009; 20:2651–60. 10.1681/ASN.2009060631 19850955 PMC2794228

[16] Najjar SS, Scuteri A, Lakatta EG. Arterial aging: is it an immutable cardiovascular risk factor? Hypertension. 2005; 46:454–62. 10.1161/01.HYP.0000177474.06749.98 16103272

[17] Lurbe E, Redon J. Isolated systolic hypertension in young people is not spurious and should be treated: con side of the argument. Hypertension. 2016; 68:276–80. 10.1161/HYPERTENSIONAHA.116.06548 27324227

[18] McEniery CM, Yasmin, Wallace S, Maki-Petaja K, McDonnell B, Sharman JE, Retallick C, Franklin SS, Brown MJ, Lloyd RC, Cockcroft JR, Wilkinson IB, and ENIGMA Study Investigators. Increased stroke volume and aortic stiffness contribute to isolated systolic hypertension in young adults. Hypertension. 2005; 46:221–26. 10.1161/01.HYP.0000165310.84801.e0 15867140

[19] Eeftinck Schattenkerk DW, van Gorp J, Vogt L, Peters RJ, van den Born BH. Isolated systolic hypertension of the young and its association with central blood pressure in a large multi-ethnic population. The HELIUS study. Eur J Prev Cardiol. 2018; 25:1351–59. 10.1177/2047487318777430 29808754 PMC6130124

[20] Flint AC, Conell C, Ren X, Banki NM, Chan SL, Rao VA, Melles RB, Bhatt DL. Effect of systolic and diastolic blood pressure on cardiovascular outcomes. N Engl J Med. 2019; 381:243–51. 10.1056/NEJMoa1803180 31314968

[21] AlGhatrif M, Lakatta EG. The conundrum of arterial stiffness, elevated blood pressure, and aging. Curr Hypertens Rep. 2015; 17:12. 10.1007/s11906-014-0523-z 25687599 PMC4524667

[22] Bavishi C, Goel S, Messerli FH. Isolated Systolic Hypertension: An Update After SPRINT. Am J Med. 2016; 129:1251–58. 10.1016/j.amjmed.2016.08.032 27639873

[23] Chobanian AV. Clinical practice. Isolated systolic hypertension in the elderly. N Engl J Med. 2007; 357:789–96. 10.1056/NEJMcp071137 17715411

[24] Pini R, Cavallini MC, Bencini F, Silvestrini G, Tonon E, De Alfieri W, Marchionni N, Di Bari M, Devereux RB, Masotti G, Roman MJ. Cardiovascular remodeling is greater in isolated systolic hypertension than in diastolic hypertension in older adults: the Insufficienza Cardiaca negli Anziani Residenti (ICARE) a Dicomano Study. J Am Coll Cardiol. 2002; 40:1283–89. 10.1016/s0735-1097(02)02159-9 12383576

[25] Papademetriou V, Devereux RB, Narayan P, Wachtell K, Bella JN, Gerdts E, Chrysant SG, Dahlof B. Similar effects of isolated systolic and combined hypertension on left ventricular geometry and function. Am J Hypertens. 2001; 14:768–74. 10.1016/S0895-7061(01)01292-4 11497192

[26] Paoletti E, Bellino D, Cassottana P, Rolla D, Cannella G. Left ventricular hypertrophy in nondiabetic predialysis CKD. Am J Kidney Dis. 2005; 46:320–27. 10.1053/j.ajkd.2005.04.031 16112052

[27] Mancia G, Giannattasio C. Diagnostic and therapeutic problems of isolated systolic hypertension. J Hypertens. 2015; 33:33–43. 10.1097/HJH.0000000000000424 25426565

[28] O’Rourke MF, Hashimoto J. Mechanical factors in arterial aging: a clinical perspective. J Am Coll Cardiol. 2007; 50:1–13. 10.1016/j.jacc.2006.12.050 17601538

[29] London GM. Arterial stiffness in chronic kidney disease and end-stage renal disease. Blood Purif. 2018; 45:154–58. 10.1159/000485146 29478047

[30] Bakris GL, Williams M, Dworkin L, Elliott WJ, Epstein M, Toto R, Tuttle K, Douglas J, Hsueh W, Sowers J. Preserving renal function in adults with hypertension and diabetes: a consensus approach. National Kidney Foundation Hypertension and Diabetes Executive Committees Working Group. Am J Kidney Dis. 2000; 36:646–61. 10.1053/ajkd.2000.16225 10977801

[31] Klag MJ, Whelton PK, Randall BL, Neaton JD, Brancati FL, Ford CE, Shulman NB, Stamler J. Blood pressure and end-stage renal disease in men. N Engl J Med. 1996; 334:13–18. 10.1056/NEJM199601043340103 7494564

[32] Ford ML, Tomlinson LA, Chapman TP, Rajkumar C, Holt SG. Aortic stiffness is independently associated with rate of renal function decline in chronic kidney disease stages 3 and 4. Hypertension. 2010; 55:1110–15. 10.1161/HYPERTENSIONAHA.109.143024 20212269

[33] Bakris GL, Weir MR, Shanifar S, Zhang Z, Douglas J, van Dijk DJ, Brenner BM, and RENAAL Study Group. Effects of blood pressure level on progression of diabetic nephropathy: results from the RENAAL study. Arch Intern Med. 2003; 163:1555–65. 10.1001/archinte.163.13.1555 12860578

[34] Verhave JC, Fesler P, du Cailar G, Ribstein J, Safar ME, Mimran A. Elevated pulse pressure is associated with low renal function in elderly patients with isolated systolic hypertension. Hypertension. 2005; 45:586–91. 10.1161/01.HYP.0000158843.60830.cf 15738348

[35] Williams B, Mancia G, Spiering W, Agabiti Rosei E, Azizi M, Burnier M, Clement DL, Coca A, de Simone G, Dominiczak A, Kahan T, Mahfoud F, Redon J, et al, and ESC Scientific Document Group. 2018 ESC/ESH guidelines for the management of arterial hypertension. Eur Heart J. 2018; 39:3021–104. 10.1093/eurheartj/ehy339 30165516

[36] Feitosa AD, Mota-Gomes MA, Barroso WS, Miranda RD, Barbosa EC, Pedrosa RP, Oliveira PC, Feltosa CL, Brandão AA, Lima-Filho JL, Sposito AC, Coca A, Nadruz W Jr. Relationship between office isolated systolic or diastolic hypertension and white-coat hypertension across the age spectrum: a home blood pressure study. J Hypertens. 2020; 38:663–70. 10.1097/HJH.0000000000002320 31790056

[37] O’Seaghdha CM, Hwang SJ, Upadhyay A, Meigs JB, Fox CS. Predictors of incident albuminuria in the Framingham Offspring cohort. Am J Kidney Dis. 2010; 56:852–60. 10.1053/j.ajkd.2010.04.013 20599306 PMC3198053

[38] Franklin SS, Pio JR, Wong ND, Larson MG, Leip EP, Vasan RS, Levy D. Predictors of new-onset diastolic and systolic hypertension: the Framingham Heart Study. Circulation. 2005; 111:1121–27. 10.1161/01.CIR.0000157159.39889.EC 15723980

[39] Li X, Lian R, Zhu Y, Ke J, Li M, Wang C, Lou T. Masked morning hypertension correlated with target organ damage in non-dialysis patients with chronic kidney disease. J Hypertens. 2020; 38:1794–801. 10.1097/HJH.0000000000002461 32694329

[40] Wang C, Deng WJ, Gong WY, Zhang J, Tang H, Peng H, Zhang QZ, Ye ZC, Lou T. High prevalence of isolated nocturnal hypertension in Chinese patients with chronic kidney disease. J Am Heart Assoc. 2015; 4:e002025. 10.1161/JAHA.115.002025 26089178 PMC4599541

[41] Kario K, Shin J, Chen CH, Buranakitjaroen P, Chia YC, Divinagracia R, Nailes J, Hoshide S, Siddique S, Sison J, Soenarta AA, Sogunuru GP, Tay JC, et al. Expert panel consensus recommendations for ambulatory blood pressure monitoring in Asia: the HOPE Asia network. J Clin Hypertens (Greenwich). 2019; 21:1250–83. 10.1111/jch.13652 31532913 PMC8030405

[42] Lang RM, Badano LP, Mor-Avi V, Afilalo J, Armstrong A, Ernande L, Flachskampf FA, Foster E, Goldstein SA, Kuznetsova T, Lancellotti P, Muraru D, Picard MH, et al. Recommendations for cardiac chamber quantification by echocardiography in adults: an update from the American Society of Echocardiography and the European Association of Cardiovascular Imaging. J Am Soc Echocardiogr. 2015; 28:1–39.e14. 10.1016/j.echo.2014.10.003 25559473

[43] Lang RM, Bierig M, Devereux RB, Flachskampf FA, Foster E, Pellikka PA, Picard MH, Roman MJ, Seward J, Shanewise J, Solomon S, Spencer KT, St John Sutton M, Stewart W. Recommendations for chamber quantification. Eur J Echocardiogr. 2006; 7:79–108. 10.1016/j.euje.2005.12.014 16458610

