# Supplementary material for: Effect of age and isolated systolic or diastolic hypertension on target organ damage in non-dialysis patients with chronic kidney disease
Source: Aging (Albany NY). 2021 Feb 22;13(4):6144–55. doi: 10.18632/aging.202609 (PMC7950225; doi:10.18632/aging.202609)
Supplement: Supplementary Table 1 [file aging-13-202609-s001.pdf]

SUPPLEMENTARY TABLE

Supplementary Table 1. Univariate linear regression analysis of age and Log (albumin creatinine ratio) (ACR) in CKD patients with different etiologies.

	Age (per 1 year)		Age (0= age<60year, 1= age≥ 60year)	
	**Beta coefficients**	***P* value**	**Beta coefficients**	***P* value**
Primary glomerulonephritis (1059)	0.001(-0.002~0.003)	0.485	0.115(0.017~0.213)	0.022
Diabetic nephropathy (252)	-0.006(-0.014~0.002)	0.149	-0.190(-0.364~-0.016)	0.032
Hypertensive nephropathy (124)	-0.012(-0.024~0.001)	0.060	-0.275(-0.573~0.023)	0.070
Other causes (574)	-0.009(-0.015~-0.003)	0.003	-0.002(-0.202~0.199)	0.988
